# Fractal and Machine Learning Analyses of MALDI-TOF Mass Spectrometry Data in Glioblastoma

**DOI:** 10.34133/csbj.0169

**Published:** 2026-08-04

**Authors:** Lucas C. Lazari, Ghasem Azemi, Carlo Russo, Livia Rosa-Fernandes, Sueli M. Oba-Shinjo, Suely K. N. Marie, Antonio Di Ieva, Giuseppe Palmisano

**Affiliations:** ^1^ GlycoProteomics Laboratory, Department of Parasitology, ICB, University of São Paulo, São Paulo, Brazil.; ^2^ Computational NeuroSurgery (CNS) Lab, Faculty of Medicine, Health and Human Sciences, Macquarie Medical School, Macquarie University, Sydney, NSW, Australia.; ^3^ Motor Neuron Disease Research Centre, Macquarie Medical School, Macquarie University, Sydney, NSW, Australia.; ^4^ Laboratory of Molecular and Cellular Biology, Lim 15, Department of Neurology, Faculdade de Medicina USP, São Paulo, Brazil.

## Abstract

Data preprocessing is a critical step in the analysis of matrix-assisted laser desorption/ionization–time-of-flight mass spectrometry (MALDI-TOF MS) spectra for machine learning applications, typically involving steps such as spectra trimming, baseline correction, smoothing, transformation, and peak picking or spectral binning. While traditional approaches focus on protein/peptide peaks as features, this study explores a novel method of feature extraction by treating MALDI-TOF spectra as one-dimensional signal array further processed as time-series data. This study investigates the use of computational fractal-based analysis to assess the complexity of MALDI-TOF spectra. Fractal analysis, previously successful in glioblastoma diagnosis using magnetic resonance imaging, was applied here to proteomics data. By treating each MALDI spectrum as a time series and calculating its fractal dimension using various algorithms, machine learning models were trained to differentiate between glioblastoma patients and controls. We demonstrate that fractal dimensions are sufficient to obtain accurate models for glioblastoma diagnosis, despite still underperforming when compared to the traditional feature extraction method. We also show that fractals can be used as support features to increase model performance. This work highlights the potential and limitations of fractal analysis in proteomics, offering a new perspective for disease diagnosis and broadening the available computational tools for data analysis in mass spectrometry.

## Introduction

Data preprocessing is an essential step for analysis with matrix-assisted laser desorption/ionization–time-of-flight mass spectrometry (MALDI-TOF MS) spectra for machine learning applications. The preprocessing steps usually consist of spectra trimming, baseline correction, smoothing, transformation, and peak picking [1]. Conversely, the peak picking step can be replaced by spectral binning, in this way the features are no longer protein/peptides *m*/*z* (mass/charge ratio) peaks, rather the whole spectra [2]. We demonstrate in this work a new approach to extract features from MALDI-TOF data by using time-series data analysis tools.

The main question was whether or not a MALDI spectrum could be considered a time-series data. The mass-to-charge ratios can be converted to time, since they are calculated based on the time of flight of the molecules before they reach the detector. This is different from evaluating the proteomic profile of a biological sample collected in a longitudinal manner, such as sera collected at different time points from the same patient. With this limitation in mind, we considered a MALDI-TOF spectrum as a one-dimensional (1D) ordered signal that could be processed as a time series.

In this context, working with the spectrum as an ordered signal allows the use of different data analysis techniques. Among these techniques, computational fractal-based analysis provides potentially useful markers for the assessment of time series’ complexity. It is important to note that fractal analysis can be implemented in MS data even if it is not considered a time series; however, we aimed to explore whether fractal dimension (FD) calculation methods specifically suited for time-series data could be applied to MS data.

One of the most used parameters in fractal geometry is the FD, used to characterize the self-similarity and repeating patterns across different scales through non-Euclidean dimensions of an object [3]. The FD of an object increases with its structural complexity [4]. Fractal analysis has already been successfully employed in glioblastoma (GBM) diagnosis and prognosis using imaging techniques such as magnetic resonance imaging, demonstrating that high-grade tumors tend to have structural and angioarchitectural higher FD values than normal brain or lower grade tumors [5–7]. Fractal analysis can also be applied to time series data, where the time series pattern may exhibit fractal scaling properties [8]. Regarding proteomics data, there is no report in the literature involving the use of FD for the extraction of spectral features.

Due to the lack of research involving FD in mass spectrometry, we decided to explore this topic using MALDI-TOF MS data in GBM diagnosis. For that, the FD metrics of each MALDI-TOF MS spectrum were calculated using different algorithms. These measurements were then used to train machine learning models to distinguish between GBM patients and healthy controls. We demonstrate the potential and limitations of this approach in MS-based proteomics, highlighting its possible application across different diseases and in different disease contexts.

## Methods

**Fig. 1.**
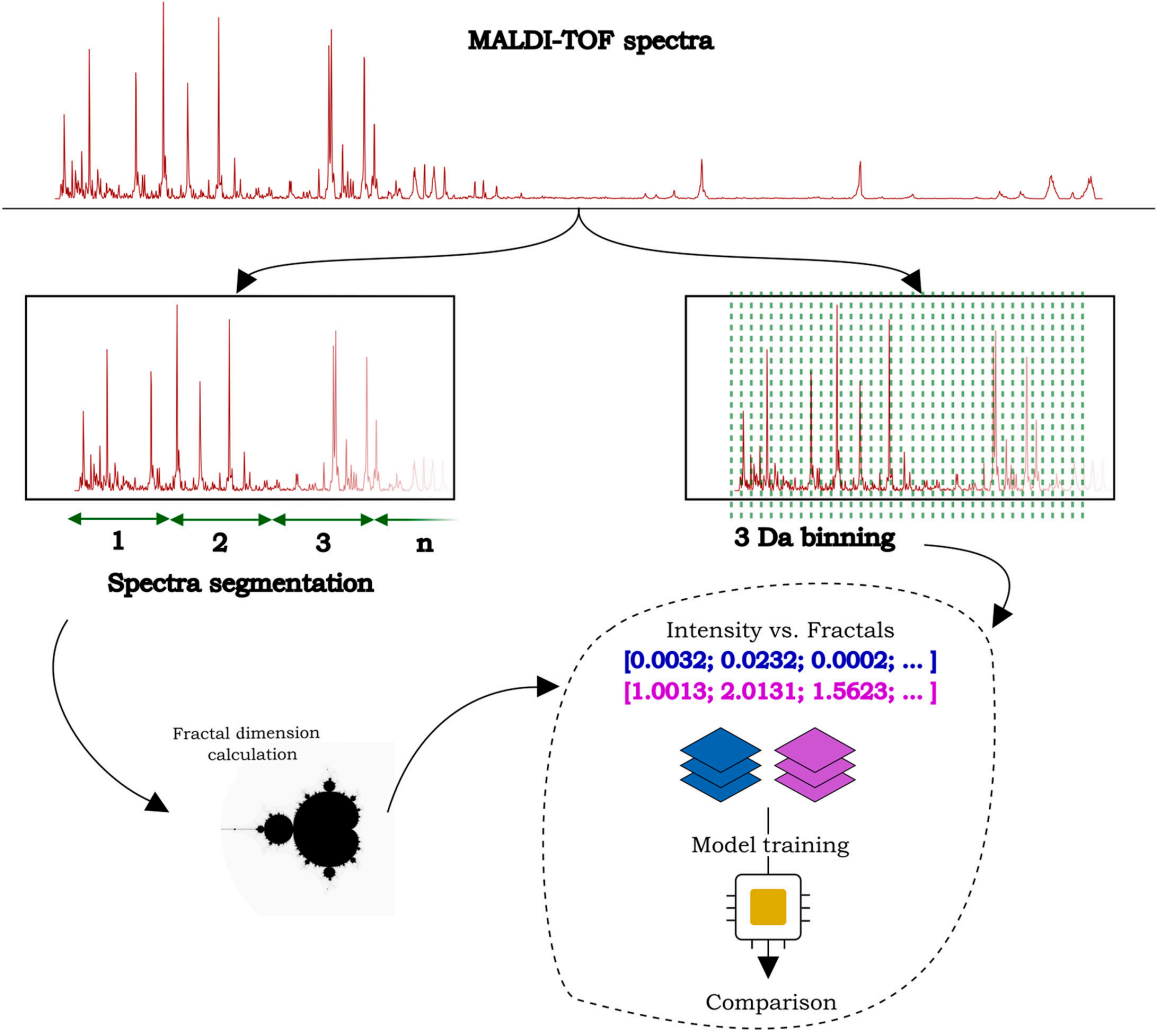
Representation of the methods to perform fractal analysis in mass spectrometry data. The methodology consists of 2 approaches: a classical approach to generate features by using the whole binned spectra and a newly developed method based on features generated by calculating the FDs of the segmented spectra.

In this study, we evaluated the utility of FD for classifying GBM patients by comparing models trained with FDs to models trained with binned spectra. For classification purposes, we used the entire spectra of the samples. The binning procedure was intended to reduce the effect of intersample variability (such as peak shifts) and to standardize all samples to the same dimensionality. However, in our case, all samples already had the same dimensionality. The model training was performed in a computer with an AMD Ryzen 5 5600 CPU. A graphical abstract about the methods employed in this work is presented in Fig. 1.

### Dataset overview

The MALDI-TOF MS spectra from GBM and control patients were obtained from PRIDE database (accession number: PXD060922) from our previous work on GBM proteomics [9]. In the referenced study, samples were analyzed using a MALDI-TOF Autoflex Speed Smartbeam mass spectrometer (Bruker Daltonics), operated via FlexControl software (version 3.3; Bruker Daltonics). Data acquisition was performed in positive linear mode with the following settings: laser frequency of 500 Hz, extraction delay of 390 ns, ion source 1 at 19.5 kV, ion source 2 at 18.4 kV, lens voltage of 8.5 kV, and a mass range spanning 2,000 to 20,000 Da. Spectra were collected in automatic mode to minimize operator bias. For each sample, a total of 2,500 laser shots were accumulated in increments of 500 shots. External calibration was carried out using Protein Calibration Standard I (Bruker Daltonics), consisting of Insulin ([M+H]+ = 5,734.52), Cytochrome C ([M+2H]2+ = 6,181.05), Myoglobin ([M+2H]2+ = 8,476.66), Ubiquitin I ([M+H]+ = 8,565.76), Cytochrome C ([M+H]+ = 12,360.97), and Myoglobin ([M+H]+ = 16,952.31).

A total of 283 files were retrieved from the database. The downloaded files included 211 oncological patients, 62.44% of whom were male and 37.56% female, with ages ranging from 0.5 to 69 years. Most patients (78.68%) were diagnosed with GBM, while 11.17% had grade 2 (AG2) tumors, and 10.15% had grade 3 (AG3) tumors. The control group consisted of 72 individuals, 65.28 % female and 34.72% male, with ages ranging from 28 to 86 years (Table 1). For this study, all GBM samples, regardless of the grade, were considered tumor samples for the classification task.

**Table 1.** Cohort summary, describing the distribution of age, sex, and tumor grade of the patients involved in this study

	Age (years)	Sex	Grade
Oncological patients	Min - 0.5	F - 37.56%	GBM - 78.68%
	Max - 69	M - 62.44%	AG2 - 11.17%
	Mean - 49.77		AG3 - 10.15%
			
Controls	Min - 28	F - 65.28%	-
	Max - 86	M - 34.72%	-
	Mean - 56.19		-

### Spectra file acquisition and preprocessing

The mzML files were preprocessed in R using the MALDIQuant package [10]. All spectra were first smoothed using the “SavitzkyGolay” method with a half window size of 10. Baseline correction was then performed with the TopHat method, and finally, the intensities were normalized to the total ion current. Although all spectra were processed concurrently, each spectrum underwent the preprocessing procedures individually, ensuring that no data leakage occurred during the preprocessing step.

### FD calculation

The Petrosian, Katz, Higuchi, and Detrended fluctuation FDs were calculated using the AntroPy library [11]. The MALDI-TOF MS spectra was treated as a 1D ordered signal, using the intensities from 2,000 *m*/*z* to 20,000 *m*/*z*. The FD values were calculated using the complete spectra, and the spectra were divided in windows of fixed size (600, 800, 1,000, 1,200, 1,400, and 1,600 data points), meaning that the intensity vector was divided in intervals of fixed size and all FDs were calculated within that interval. This approach generates more features as the window size decreases. All parameters from the AntroPy package were set to their default values (higuchi kmax = 10).

Higuchi, Katz, Petrosian, and detrended fluctuation analysis quantify signal complexity through different definitions of scaling. Higuchi FD estimates how the length of a curve changes with sampling interval by constructing multiple sub-series at different step sizes, making it sensitive to fine-scale irregularities [12]. Katz FD uses a geometric ratio between the total curve length and the maximum distance from the starting point, yielding a fast but more heuristic measure of complexity [13]. Petrosian FD relies on the number of sign changes in the signal (or its derivative), effectively capturing roughness via zero-crossing structure and being relatively robust to noise but less granular [14]. Detrended fluctuation analysis instead evaluates how fluctuations scale with window size after removing local trends, producing a scaling exponent related to long-range correlations rather than purely geometric roughness [14].

Applying these time-series analysis methods to MALDI-TOF spectra can be done because the data can be treated as an ordered 1D signal over the *m*/*z* axis, where intensity varies across scales (peak widths, baseline variations, noise). These estimators do not require temporal dynamics; they only assume an ordered sequence with scale-dependent structure. In this context, the resulting FD should not be interpreted as a physical fractal property of the sample, but as a quantitative descriptor of spectral complexity and irregularity, capturing features such as peak density, sharpness, and multi-scale variability. Therefore, these metrics serve as compact, model-agnostic features that summarize the morphology of spectra and can possibly be used by machine learning models for classification.

### Spectra binning

Using the method described by Weis et al. [2], the binning procedure was performed and the preprocessed spectra were partitioned into nonoverlapping bins of 3 *m*/*z*. The intensities within each bin were summed. This procedure not only standardizes the data but also greatly reduces the dimensionality of the spectrum.

### Machine learning for classification

**Fig. 2.**
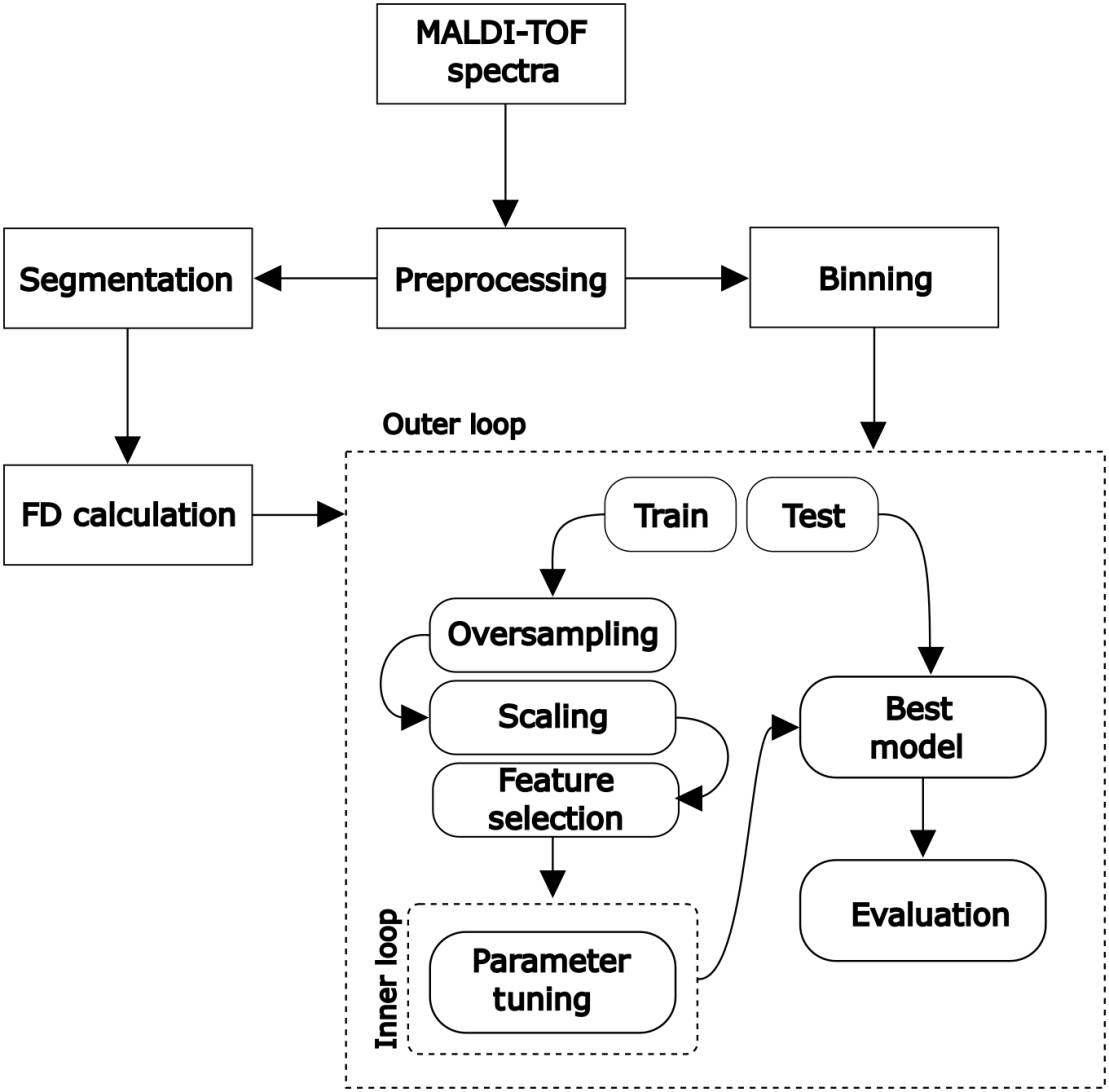
Pipeline used for model training and evaluation of FD in mass spectrometry. An outer loop is used to separate the data into train and test sets. Then, the training set is processed and used in an inner loop for hyperparameter tuning through a 3-fold cross-validation. The best model is then selected and evaluated using the hold-out test set. The scaling and feature selection procedures are performed only in the training set and then passed to the test set to avoid overfitting.

The procedure for tumor classification using FDs and the binned spectra followed the same pipeline. First, the dataset was split into 5 folds of train and test sets using the stratified *k*-fold function of sklearn python library [15]; this was done to ensure that both approaches were trained using the same set of samples in each fold, thus ensuring that the results are comparable. Then, a 5-fold nested-repeated 3-fold cross-validation was performed for hyperparameter tuning and model evaluation. The pipeline used in this work for model training is shown in Fig. 2.

After selecting the best set of hyperparameters in the inner loop, the best model is evaluated using the test set; this is performed across all the dataset divided in 5 folds. The models’ performances were evaluated for the dataset with and without oversampling and with different methods for feature selection [BorutaPy [16], principal components analysis (PCA), partial least squares (PLS), receiver-operating characteristic curve (ROC), and area under the curve (AUC)]. The pipeline with highest performance that was used to generate all data in the results section was with synthetic minority over-sampling technique (SMOTE) to oversample the control class, scaling the training and test sets using the StandardScaler function from sklearn (the scaler was fit in the training set) and BorutaPy for feature selection (n_estimators = “auto”, alpha = 0.001); these procedures were performed within each fold. The resulting features were used to train 3 models (Random Forest, LightGBM, and SVM), and performance metrics including sensitivity, specificity, and balanced accuracy were evaluated. Finally, the best model was selected to run another 5-fold cross validation with Shapley Additive Explanations (SHAP library) [17] to evaluate the feature contribution; for that, we used the KernelExplainer algorithm. SHAP was selected as a model explainer tool in this case since it can be run in a wide range of models, as well as its known use in proteomics research [18–20]. The performance metrics for all other feature selection methods and the models with oversampling are available in Table S1.

Confusion matrices were created to calculate the performance metrics for each test performed; ROC and precision-recall (PR) curves were also generated. A mean spectrum was generated for each group, and the interquartile range was calculated and plotted as a shaded area in the mean spectrum. The most important features accordingly to SHAP [17] for both fractal and binned features were plotted alongside the spectrum to search for regions of interest.

Tumor grade classification was also performed; however, it did not yield any model with acceptable performance, as was reported in the original GBM paper where this dataset was retrieved from [9].

## Results

### Classification performance in varying window size

**Fig. 3.**
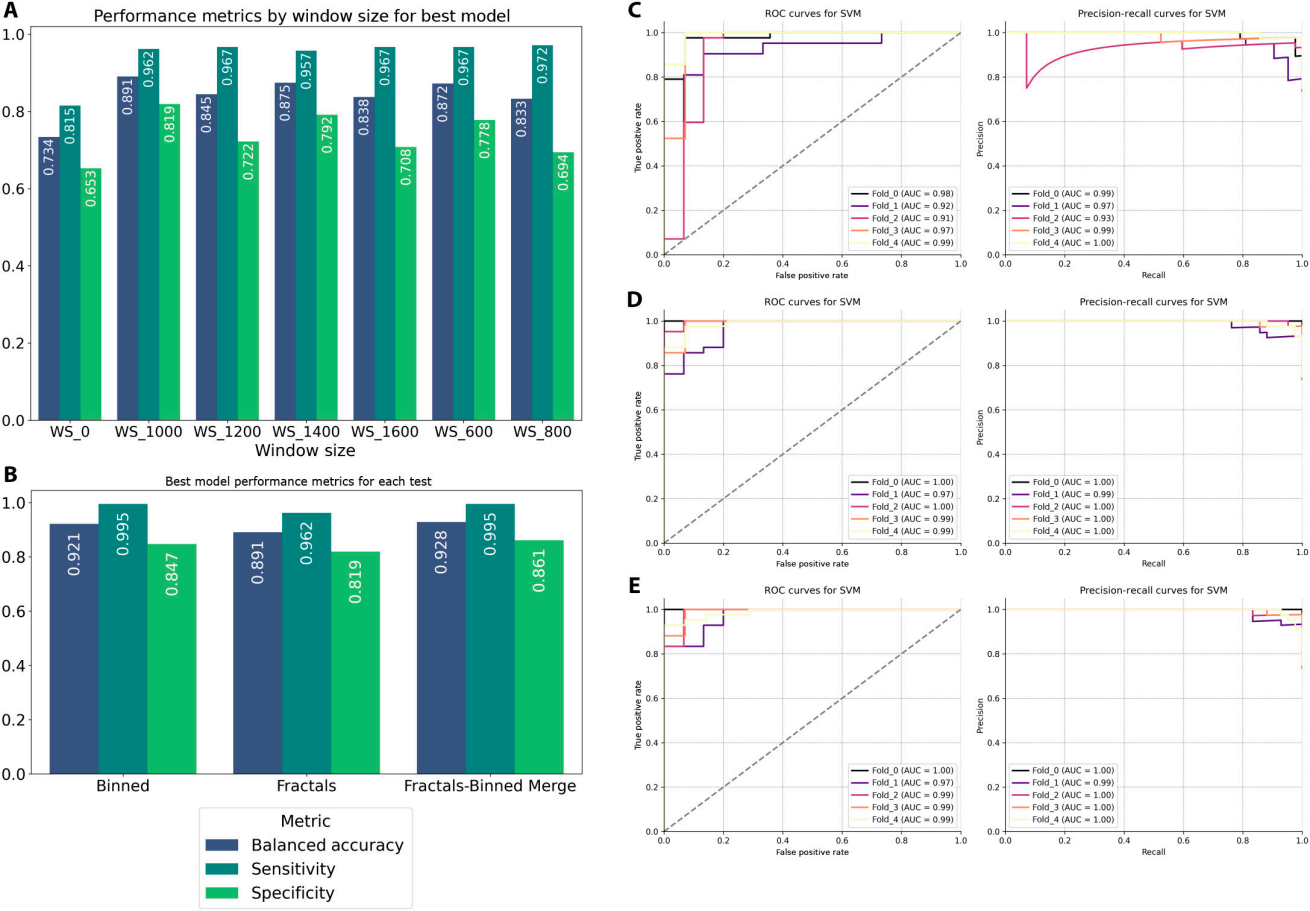
Performance of the models trained with fractal and binned data. (A) Performance metrics for the best model in each window size tested. (B) Performance metrics for the best model in each dataset tested. (C) ROC and PR curves for the best model trained with the fractal feature dataset. (D) ROC and PR curves for the best model trained with the binned dataset. (E) ROC and PR curves for the best model trained with the merged dataset.

To find the optimal window size for the classification task using FD-based features on model performance (balanced accuracy), we segmented the intensities arrays in different sizes (600, 800, 1,000, 1,200, 1,400, and 1,600 delta *m*/*z* values) and also tested the classification performance using the complete intensity array from the entire *m*/*z* range. Therefore, we searched for the hyperparameter sets and trained the 3 models (Random Forest, LightGBM, and SVM) on each window size and compared the balanced accuracy, sensitivity, and specificity. These models were selected because they are well-established machine learning algorithms and have complementary strengths; random forest (RF) provides robustness against noise and overfitting, LightGBM offers efficient gradient boosting-based learning with strong predictive performance, and SVM is particularly well suited for high-dimensional data and complex decision boundaries. The results demonstrated that the window size of 1,000 achieved the highest balanced accuracy (89.1%) using an SVM model (Fig. 3A). The lowest performance was obtained without spectrum segmentation. All models’ performances for this step are reported in Table S2.

### Comparison of fractals and binned spectral features for classification

**Fig. 4.**
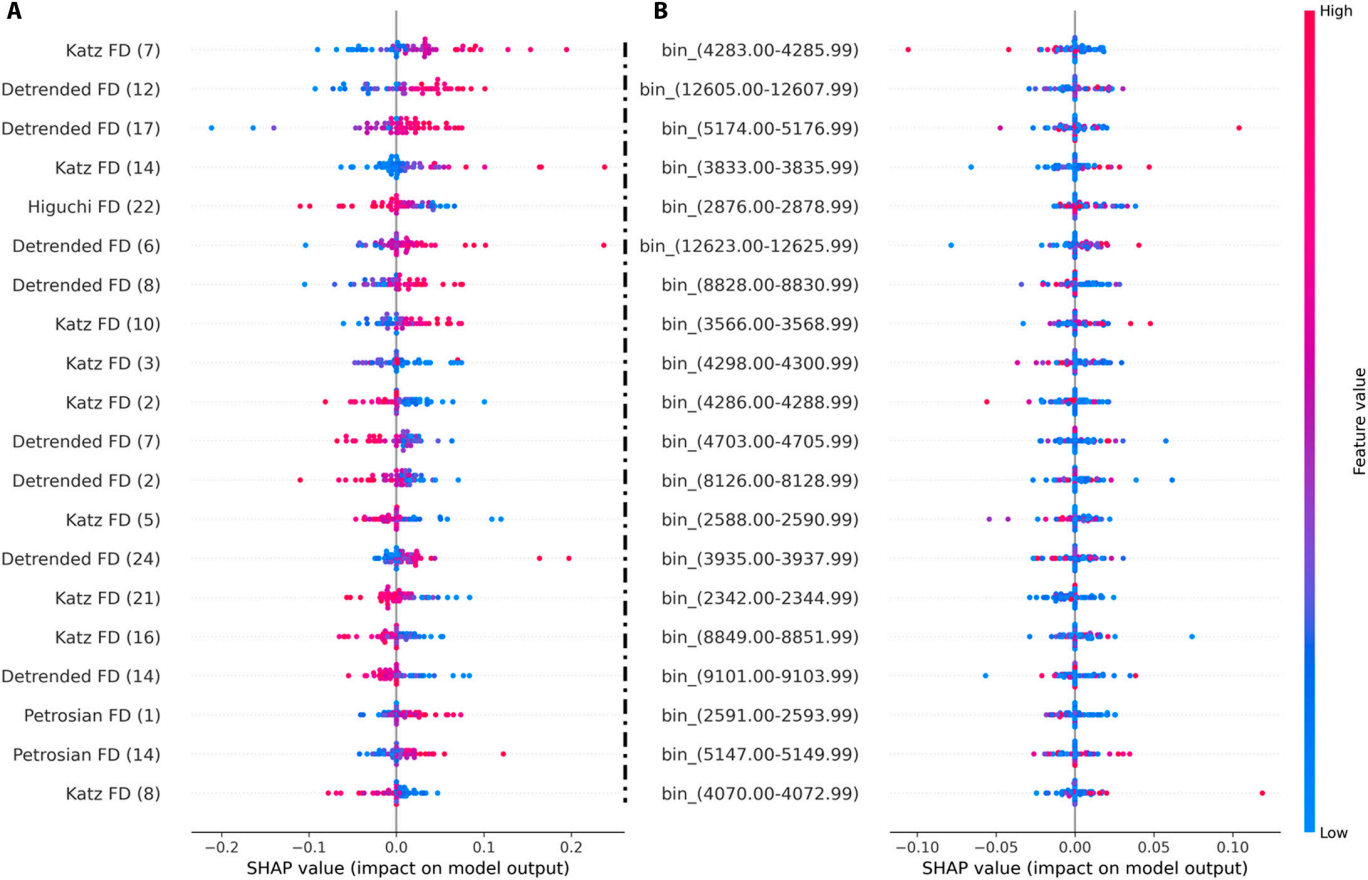
SHAP results for model explanation. (A) SHAP output for the first fold of the fractal feature dataset. (B) SHAP output for the first fold of the binned feature dataset. SHAP value indicates the impact that feature has on model output, positive values imply an impact in the classification of the positive class, while negative values imply the opposite. The color map indicates how the feature impacts model decision, e.g., if the feature has a high value and a high SHAP value, the increase of this feature is a characteristic of the positive class.

**Fig. 5.**
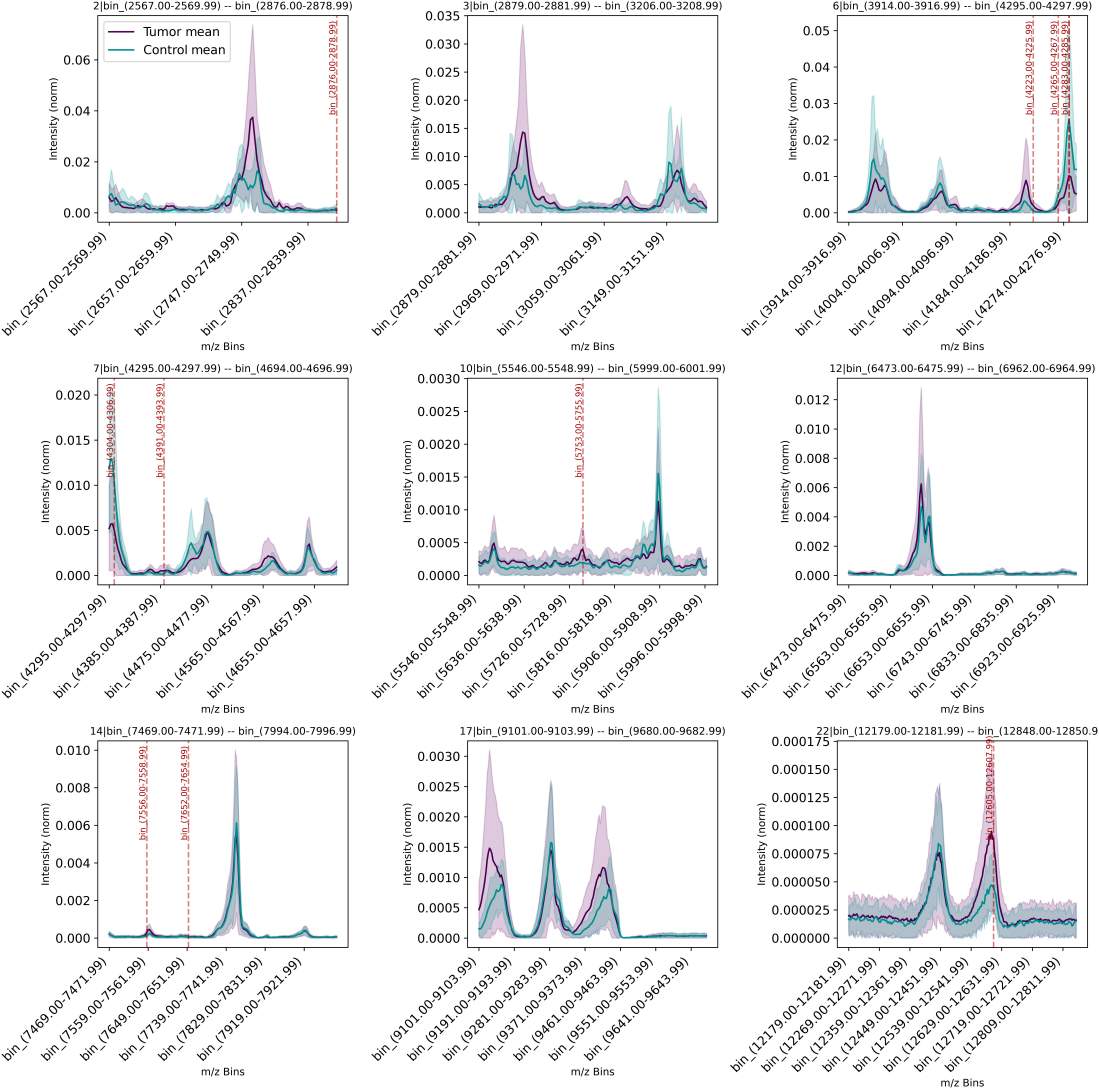
Zoomed MALDI-TOF MS spectra in the most important regions according to SHAP. On top of each plot, the first number is related to the region of the spectra that is being shown, followed by the *m*/*z* range of that window (i.e., the first zoom plot refers to region number 2; this region starts at *m*/*z* 2,567.00 and goes until *m*/*z* 2,878.99). The shaded area is the interquartile range, and the dashed red lines are relevant bins according to SHAP.

From a total of 32 intervals using the optimal window size of 1,000 data points, the total number of fractal features was 128 prior to feature selection (these features were generated by dividing the intensity array into sub-arrays of size 1,000 and calculating the FD dimensions within that sub-array), which were reduced to 73 by BorutaPy [16]. BorutaPy is a feature selection algorithm built around Random Forest; it creates “shadow features” by shuffling the original features and then compares real feature importance against these random baselines. Features consistently outperforming shadows are kept, while the rest are rejected or marked tentative. The total analysis time, from FD calculation to the 5-fold cross-validation with optimized parameters, took approximately 4 min. As described in the previous section, the highest performance obtained using fractals was obtained by the SVM model, achieving a balanced accuracy, sensitivity, and specificity of 89.1%, 96.2%, and 81.9%, respectively (Fig. 3B to E). For each fold, we used SHAP to evaluate the most important features for the classification task and how they impacted the model’s decision making (Fig. 4). We then selected the top 10 features across all folds and searched the intensity intervals in which they were calculated. This was performed to find the discriminating regions in the spectra, which could be used to detect potential biomarkers (Fig. 5). The output provided by SHAP demonstrated that different FD calculation methods in the same region of the spectra present different behaviors (Fig. 4A). For instance, detrended FD at the region of number 7 (a region here is defined as the *m*/*z* window of the spectra where this metric was calculated from; the exact *m*/*z* range for the most interesting regions is shown in Fig. 5) has a negative correlation with the classification of the positive class (GBM patients), meaning that when this value is high, the model tends to classify the individual as negative class (control). However, Katz FD in the same region had the opposite behavior (Fig. 4A). This strengthens the idea of using multiple FD calculation methods, being beneficial to capture the complex nature of MALDI-TOF MS proteomics. To evaluate this claim, we tested each FD metric individually in the classification task. The results demonstrated that none of them individually could outperform the model trained with the combination of multiple FD metrics (Table S3).

Interestingly, most of the fractal features across all folds had a clear impact for the model’s decision making, meaning that it is simpler to interpret whether the increase or decrease of a feature will impact the model’s classification of the positive class when compared to the binned spectrum (Fig. 4).

For the binned spectra, the number of features before the application of a feature selection method was 4,336 (generated by grouping the *m*/*z* into 3-Da intervals and summing their intensities), which were reduced to 470 using BorutaPy. The total analysis time in this case was approximately 16 min; however, the performance was higher than the fractal feature-based dataset. Using the binned spectra, we achieved a balanced accuracy, sensitivity, and specificity of 92.1%, 99.5%, and 84.7%, respectively (Fig. 3B). We applied SHAP in all folds to find the most important bins and to evaluate how they impact the model’s decision making. Conversely from fractal features, the binned features across all folds are smaller in magnitude, meaning that it is difficult to evaluate how the increase or decrease of a feature will impact the model’s classification of the positive class (Fig. 4B). The binned features that were present in the same region as the selected fractal features were pointed out in the plot (Fig. 5).

All models’ performances, their respective ROC and PR curves, and the best hyperparameter sets are available in the Supplementary Materials. The SHAP output for each fold for both fractal and binned features is available in the Supplementary Materials.

Following SHAP results, we extracted the top 5 features of each fold and selected the unique features as candidates for biomarkers. Figure 5 shows the unique spectra regions that contained the top 5 fractal features and the top 5 bins that fall within that region. This demonstrates that FD can be used to tune up the search for relevant regions that could be further investigated using other proteomics techniques, with the final aim of identifying proteins that might be useful as biomarkers. It is important to note that follow-up orthogonal proteomic validation is necessary for biomarker discovery.

Regions 2, 3, and 7 show a clear distinction between the groups, with the appearance of mass peaks with different intensities between the groups.

## Discussion

FDs have found substantial applications in the analysis of time series data in a wide range of fields, such as climate and medicine research [21,22]. Concepts related to fractal or non-integer dimensionality have also been explored in MALDI-TOF data, for example, through manifold dimension estimation based on scaling relationships in spectral space [23].

A MALDI-TOF MS spectrum is not typically considered time-series data, as they typically consist of sequential measurements taken at specific time interval, while MALDI-TOF spectrum represents data as intensity versus *m*/*z* ratio, reflecting the distribution of molecular masses at a single point in time, lacking the temporal sequence characteristic to time-series data; however, the spectrum can be treated as an ordered 1D signal over the *m*/*z* axis, thus allowing the usage of time-series specific data analysis tools. Apart from one work that modeled MALDI-TOF MS data as time series from spectral clustering [24], no other work has analyzed it directly using time-series data analysis tools. In our study, we investigated whether the use fractal analysis tools tailored for the study of time-series data could be directly applied to MALDI-TOF MS data.

In the present study, we re-analyzed the MALDI-TOF MS data from a GBM cohort to develop a new data processing pipeline for machine learning classification using Antropy library for fractal analysis. Although the use of MALDI-TOF MS data has already been explored in the literature [25], this work demonstrated for the first time that MALDI-TOF MS spectrum has fractal properties and the FDs of the protein profile of healthy and GBM patients differ, suggesting the existence of a GBM’s mass spectral fractal fingerprint. The use of FD as features for model training yielded a high performance in classification model, but lower when compared to the binned features. However, the merge of both feature types caused a slight increase in overall model performance. This indicates that although FDs do not outperform spectrum binning, fractal analysis can be still used as a complementary analytical tool. A clear advantage to use fractals in this case is to reduce processing time, since fractal analysis was significantly faster than spectrum binning (approximately 4 times faster), being a promising technique for the analysis of very large datasets.

SHAP analysis demonstrated that FD can also be used to search biomarkers by finding spectra regions with highly significant features. These regions of interest could be evaluated using other proteomics techniques to identify proteins in that mass range and assessing their potential as biomarkers.

The possibility of using fractal analysis in mass spectrometry data opens a new field for proteomics, where FD can be explored not only in GBM research but also in many other diseases. To this point, we found that FD can be implemented to the analysis of mass spectrometry data and can be used as complement to enhance model performance in classification tasks, with more investigation needed to assess other possible applications of this technique in proteomics. Also, this work demonstrated that time-series data analysis tools, such as Antropy used in this work, can be used to analyze and extract features from MALDI-TOF MS data (and possibly other MS data), which allows researchers to explore a wide range of time-series analytical tools.

## Conclusions

This study demonstrates that FDs of MALDI-TOF MS spectrum can be used to characterize GBM in liquid biopsies. FDs can also be used to search for regions of interest in the spectra, which could be further translated into biomarker discovery with proper orthogonal validation techniques. Although it could not outperform the models trained with binned spectra, fractals can serve as a complement to increase model performance. Since the analysis time was significantly lower, fractals could be an alternative to deal with very large datasets. This is the first report of fractals being associated with mass spectrometry data for feature extraction, and it shows an alternative path for proteomics data analysis. The methodology employed in this work could be used to study not only GBM but also many other diseases that are already being explored using MALDI-TOF data. It also opens the possibility to test FD in other mass spectrometry data types.

## Data Availability

The MALDI-TOF MS spectra used in this work were obtained from PRIDE database under the accession number: PXD060922. The script used in this work to run the analysis reported can be provided under a reasonable request.
